# Is There a Relationship between Research Sponsorship and Publication Impact? An Analysis of Funding Acknowledgments in Nanotechnology Papers

**DOI:** 10.1371/journal.pone.0117727

**Published:** 2015-02-19

**Authors:** Jue Wang, Philip Shapira

**Affiliations:** 1 Public Policy and Global Affairs Program, School of Humanities and Social Sciences, Nanyang Technological University, Singapore; 2 Manchester Institute of Innovation Research, Manchester Business School, University of Manchester, Manchester, United Kingdom; 3 School of Public Policy, Georgia Institute of Technology, Atlanta, GA, United States of America; State University of New York, Oswego, UNITED STATES

## Abstract

This study analyzes funding acknowledgments in scientific papers to investigate relationships between research sponsorship and publication impacts. We identify acknowledgments to research sponsors for nanotechnology papers published in the Web of Science during a one-year sample period. We examine the citations accrued by these papers and the journal impact factors of their publication titles. The results show that publications from grant sponsored research exhibit higher impacts in terms of both journal ranking and citation counts than research that is not grant sponsored. We discuss the method and models used, and the insights provided by this approach as well as it limitations.

## Introduction

With demands by scientists, universities, and other stakeholders to expand research and development (R&D) funding, there are also growing pressures to assess and justify the impacts of R&D expenditures. Governments typically sponsor most basic R&D and thus underwrite a significant share of the knowledge production and resulting scientific publications that are generated by national R&D investments [1]. For government agencies in many countries, public accountability is requiring more attention to the effectiveness and efficiency of public research funding. In the US, for example, the Government Performance and Result Act of 1993 [2] aims to increase the efficiency, effectiveness and accountability of federal spending, leading to the use of performance measures with identified R&D goals and outcomes. The Science of Science and Innovation Policy (SciSIP) Program of the US National Science Foundation particularly supports research on approaches to measure returns from R&D investments [3]. Similarly, the Japan Science and Technology Agency, through its Research Institute of Science and Technology for Society, has established a Program in Science of Science, Technology and Innovation Policy to assess the economic and social impacts of R&D investments [4]. While much attention is of course focused on industrial and commercial outcomes, there is widespread interest in scientific results, including both the quantity and quality of publication outputs from R&D, and this interest extends not only to the public sector but also to foundations and corporate research sponsors.

Many studies have attempted to quantify the economic return and non-economic return of investment in R&D. The former includes those measuring the impact of private R&D investment [5–9] and impact of public R&D investment [10–14] on economic growth. The latter includes studies measuring the effect of R&D funding on the amount of scientific output such as publications and patents [15–18] and the impact and quality of scientific output [19–24].

This paper seeks to extend work in the second category, on the relationships between R&D sponsorship and publication outputs. We examine funding acknowledgments reported in scientific papers to investigate the relationships between funding and research impacts. Utilizing the context information in the acknowledgment fields in publications, the approach allows for large scale analysis of funding acknowledgment variables [25–27]. This approach uses publicly-accessible data sources and, unlike many prior approaches, is not limited to any particular funding agency or research institution, which facilitates comparisons across various boundaries (institutional, sponsor, national, and discipline). While this study makes use of publications in the interdisciplinary domain of nanotechnology, the approach can readily be extended to other fields.

A two-stage regression model is used to test the effects of grant funding on research impacts. The first stage regression examines whether papers supported by grant funding sources are more likely to get published in high impact factor journals, which can be viewed as recognition by peer reviewers. The second stage regression examines whether papers with grant funding are more likely to receive attention and generate citations after being published. This can be viewed as indicating use by and diffusion to the broader scientific community. Two bibliometric indicators—journal impact factor and citation counts—are used to measure research outputs in these two stages. Both indicators are not without critiques. It is argued that these measures are proxies for quality, although it is also argued that such claims should be treated carefully due to field differences, biases in peer review, citations clubs, and the possibility of negative as well as positive types of citations [28]. Nevertheless, these two measures are frequently used as indicators for assessing the publication outputs of scientists and their research groups, by promotion and tenure committees and in studies of the performance of research institutions and nations. The use of both indicators in combination can offset some of the problems associated if they are used individually. A dataset of over 89000 nanotechnology publications is used to empirically test the model effects. The results show that publications associated with grant sponsored research do exhibit higher impacts in terms of both journal ranking and citation counts, controlling for field differences. At the same time, impacts also vary by funding sources and patterns. Our empirical results and their implications are discussed in detail later in the paper, following discussion of the existing literature and of our study hypotheses.

## Literature Review

This section of the paper reviews a range of literature that reports on prior work related to research sponsorship and publication impacts. In this process, we draw out four hypotheses to test in our subsequent large-scale empirical work. We first consider the subject hypotheses in the context of the literature, and then discuss how we operationalize the notion of publication impact.

In general, the extant literature suggests that there are connections between research funding and research outputs, although there is a nuanced debate about the nature and direction of effects. A predominant perspective is that research funding has positive impacts on research outputs. Researchers who secure extra funding may well be more determined and ambitious in their research goals and able to garner more resources (e.g. for personnel, equipment, materials, and travel) for implementing their research. In some instances, efforts to secure additional research funding may reflect departmental or institutional pressures to develop research credibility or enhance visibility. Grant proposals typically go through review processes in order to get funded—for examples, see relevant sections on peer and merit review processes promulgated by leading funding agencies such as the UK Engineering and Physical Sciences Research Council (EPSRC) [29], the US National Institutes of Health (NIH) [30] and the US National Science Foundation (NSF) [31]. Funding agencies may have their own research agendas, and target proposals that fit these priorities, and they may also have open submission rounds where researchers propose new research topics. In either case, peer review processes aim to filter out weaker research proposals and reward more promising research ideas. On the other hand, peer review can be flawed, biased or conservative, and the administrative time and costs associated with proposing and managing grant sponsored research may distract from scientific advancement [32]. One US study of faculty workloads found that more than two-fifths of time allocated to federally funded research was spent on pre- and post-award administrative activities rather than active research, with variations across institutions and disciplines [33]. Research groups with better administrative coordination or more institutional support may be less distracted by grant award transaction costs. However, such issues may be less apparent when research is block-supported, i.e. where scientists receive support for their research work on a regular basis, usually from internally-allocated resources, without the need to compete for external sources of grant funds. Block-supported research may allow researchers the flexibility and security of exploring riskier new ideas—or, conversely, such research might become staid without the stimulus or requirement of justifying new research ideas to external reviewers.

The empirical evidence to date on the effects of different modes of sponsoring research is mixed. For example, a study of NIH-supported researchers shows that funded publications appear to have higher citations than average (including non-grant sponsored research and presumably block-supported) publications [22]. Similar findings were reported in a study of researchers in an Australian university [23] and a study of researchers funded by the National Cancer Institute of Canada [24], where grant funded publications appear to have higher citations than non-grant sponsored research papers. By contrast, Harter and Hooten [34] found that articles published in the Journal of the American Society for Information Science (JASIS) exhibit no difference in citation counts with regard to their funding status. This result was confirmed in their subsequent study which expanded the scope of JASIS articles under study to additional years [20]. Similarly, Cronin and Shaw [19] in their study of four information science journals found no significant difference in citations between grant sponsored and non-grant sponsored research articles.

The mixed results found in previous studies can be attributed partly to different subjects under study and partly to differences in the analytical methods used. More specifically, subjects in these studies were limited in sample size and varied from publications by researchers funded by a particular agency [22] [24], to publications by researchers in a particular institution [23], and to articles from selected journals [19–20] [34]. The inherent variances in the characteristics of these subjects may lead to observed differences in the relationship between funding status and citation. Additionally, these studies used different analytical approaches. Harter and Hooten employed a correlation test between funding status and citation counts [20] [34]. Cronin and Shaw [19], Trochim et al. [22] and Campbell et al. [24] compared the difference of means between citation of funded and non-funded publications. A major problem with both correlation tests and comparison of means is that neither control for other factors affecting citations so as to isolate the impact of funding. Sandstrom [23] undertook a regression analysis with variables including several grant aspects as independent variables and citation as a dependent variable. However, these regression results need to be interpreted with caution due to the unclear causal effect direction between funding and citation in the dataset as noted by the author.

In short, while these prior studies offer insights, there remains considerable scope (and need) to advance work on the relationships between research sponsorship and publication impact and to introduce appropriate controls. The recent availability of large-scale information on funding acknowledgments in scientific research papers allows us to initiate a further and more extensive examination of this question. In this paper, we investigate four straightforward yet still critical hypotheses. To start, we examine the funding status of publications and explore if grant sponsored research receives more attention and has higher impact. By “grant sponsored research”, we mean research that explicitly acknowledges a research sponsor or grant award as providing support to the research work reported in a paper. In contrast, “non-grant sponsored research” refers to papers without funding acknowledgment. The research reported in such papers may indeed not be explicitly funded although in most cases it is likely to be block-supported by internal institutional resources. As outside research grants are often competitive and typically require external peer-review, low-quality research proposals should be screened out. It is thus plausible to conjecture that grant sponsored research, taken as a whole, will be of better quality compared with research that is not sponsored or is block-supported without going through competitive external review processes. This leads us to the initial hypothesis (H1) that grant sponsored research will be associated with higher publication impacts.

Grant sponsored research publications may have multiple funding sources. In some cases, it is the result of joint solicitation from funding agencies. For example, the National Institute of General Medical Sciences (NIGMS) and the National Science Foundation’s Division of Mathematical Sciences (NSF/DMS) jointly call for proposals titled “Research at the Interface of the Biological and Mathematical Sciences.”[35] In other cases, the publication is based on a research project funded by different agencies at different stages. However, the majority of papers which acknowledge co-funding are the product of intellectual collaboration between authors from different organizations/countries with separate funding support. These authors report the particular funding sources brought together to support the collaboration. Indeed, the relationship between co-authorship and co-funding is found to be positive. As such, a question arises whether single or multiple funding sources will achieve higher research impacts. Arguably, research with multiple funding sources has gone through more stages of review, and thus might have higher quality. Alternatively, more funding sources might require more attention to administration and coordination, which could distract from the research effort. To test, we put forward a second hypothesis (H2): research that receives sponsorship from a greater number of funding sources will have higher publication impacts.

International collaboration has been an increasing pattern in the scientific world. Not only are scientists from different countries working together, funding agencies are also seeking cooperation with foreign partners in supporting the advance of science. The US NSF recognizes the importance of international science and engineering partnership and suggests using it as a tool to address global challenges [36]. The European Commission Framework Programme intentionally requires that the proposal should be comprised of teams from multiple countries. International research groups are believed to have stronger scientific power because of their access to additional resources [37]. Narin et al. [38] also suggested a self-selection effect as scientists doing well in research are more likely to travel and co-author papers internationally. These observations support the proposition that research which involves international collaboration will tend to have higher impact. Similarly, publications with international co-funding support will also exhibit higher impact. This suggests a third hypothesis (H3): research that receives grant support from multiple countries will have higher publication impacts.

We further posit that leading countries in key scientific fields have particular research capabilities and the potential to identify and support emerging topics that can then more broadly influence future scientific directions. Research areas funded by leading countries not only direct the research interests of scientists but also influence funding priorities of other countries. Hence, we anticipate that research funded by leading countries will receive more attention in the field. This leads to our fourth hypothesis (H4): research receiving grant funding from leading countries in a scientific domain will tend to have higher publication impacts.

Two indicators are used to measure the publication impact of research: citation counts and journal impact factors. Citation counts are often used as an indicator for the impact and diffusion of research. Citations map the intellectual and knowledge linkages between the source article and reference article. High citation counts have been positively correlated with other recognized impact indicators such as peer assessment and honorific awards [28]. The frequency of a paper being cited shows that other researchers have recognized it. We fully recognize that the use of citation counts as a measure of research quality is subject to well-known limitations, such as critical citations, self-citations, and field variance [39].

The journal impact factor reflects the average citation performance of a journal. It was proposed by Garfield [40] and is used in the Journal Citation Report (JCR) [41] published by Thomson Reuters to indicate the relative ranking of journals. The impact factor is calculated as the ratio of citations received by the journal in a particular year to the number of publications in that journal in the previous two years. No impact factor is reported for journals that are newly indexed in JCR or that do not publish regularly. A journal with a higher impact factor suggests that the journal is of more prominent in its field as a source of reference knowledge and attracts papers which are more likely, on average, to be well-cited (recognizing that not all papers in journals with high impact factors receive citations). There is critical debate about using the impact factor to measure journal quality [42]. For example, the impact factor is discipline dependent where some disciplines have much more citations than other disciplines due to field size or differential propensity to cite. Additionally, journals with more review articles tend to receive more citations.

## Data and Model

The study uses the funding acknowledgment analysis approach proposed by Wang and Shapira [26] to detect the linkage between funding and research output. The acknowledgment section of a publication provides information on the financial and intellectual support received by the authors, where the names of funding organizations and often the grant numbers are specified. The acknowledgment section of the Thomson Reuters Web of Science (WoS) publication index allows for analysis of funding and publications at a large scale.

The analysis uses data of nanotechnology publications over the one year period August 2008 – July 2009. August 2008 represents the first month that funding acknowledgments were available (at the time of our analysis) in WoS publication records. We extracted publications that recorded publication year and month for our study time period (inclusive) from a global nanotechnology publication database that was developed by the Nanotechnology Research and Innovation Systems Assessment Group at Georgia Institute of Technology (Georgia Tech), Atlanta, USA. Nanotechnology is a broad domain of research at the nanoscale (1–100 nanometers) involving multiple fields including engineering, physics, chemistry, microscopy, materials, electronics, and biology. Details of the definition of nanotechnology and the search strategy are contained in Porter et al. [43]. After excluding publications with missing journal impact factor information, the dataset used in this study contains 89,605 nanotechnology publications, with 67% reporting funding acknowledgment information (Table 1). Where funding acknowledgment is not included, this is primarily because the research was funded internally or unsponsored but using available resources. There is also the possibility of unintentional or intentional neglect to acknowledge funding [44]. We judge that this does not pose a serious threat to our analysis as it is frequently (and increasingly) mandatory for researchers funded by public and other research sponsors to disclose funding sources in resulting publications. Empirical examination has found a strong correlation between grant data and publication funding acknowledgment [45]. The share of publications reporting funding acknowledgment is also comparable with other studies [26] [27]. A quarter of the publications have a single funding source and 42% have multiple funding sources. Over half of the publications receive funding from only one country (Table 2). Funding from two or more countries supports around 11% of publications. As funding arrangements can be complicated and varied in how they are reported in the acknowledgment text, with differing acronyms and misspelling of funding agencies, a large amount of work was required to text mine, clean, match and validate the data [26].

**Table 1:** Distribution of funding sources.

Number of funding sources	Frequency	Percent
0	29,403	32.81%
1	22,497	25.11%
2	18,062	20.16%
3	10,448	11.66%
4	5,075	5.66%
5	2,274	2.54%
6	982	1.1%
7	458	0.51%
8	214	0.24%
9	89	0.1%
10 or more	103	0.12%
Total	89,605	100%

Source: Analysis of nanotechnology papers, published worldwide August 2008-July 2009, and indexed in the Web of Science (see text for added details).

**Table 2:** Distribution of funding countries.

Number of funding countries	Frequency	Percent
0	29,403	32.81%
1	50,600	56.47%
2	8,092	9.03%
3	1,281	1.43%
4	194	0.22%
5 or more	35	0.04%
Total	89,605	100%

Source: Analysis of nanotechnology papers, published worldwide August 2008-July 2009, and indexed in the Web of Science (see text for added details).

In our analysis, two dependent variables are used: impact factor (*IMPACT_FACTOR*) and citation (*CITATION*). Both impact factor and citation indicate impact but in different ways. The impact factor shows the broad relative citation ranking of a journal and in our case it measures acceptance by peer reviewers, while citation measures recognition by the research field. It is possible that individual articles in highly-ranked (“good”) journals do not receive many citations while some articles in less-well ranked journals can be cited extensively. The impact factor is also used as a control variable in the citation model to test whether articles in good journals receive more citations. The journal impact factor is derived from the WoS Journal Citation Reports (JCR) [41]. JCR impact factors were sought for the journal titles of all publications in our nanotechnology data set. Impact factors (2009) were found for 3686 or 93.3% of the 3952 titles, covering 89605 or 97.8% of the papers. For the titles in our nanotechnology publications database, the median journal impact factor was 2.7, with a mean of 3.2. Data on forward citations is also retrieved from WoS and counted as of December 2010. This should be regarded as an early measure of citations, representing those garnered between 17 to 28 months following journal publication. Around 19% of articles received no citations from publication to this date. Overall, the average citation count is 4.7 and the median is 3. Since citation is a count variable, a negative binomial regression is used to model the relationship between funding and citation. The negative binomial regression is good for modeling over-dispersed count outcome variables [46].

Variables on author numbers, affiliations, and countries, as well as fields of research are included as control variables to isolate the impact of funding. Collaborative work, academic affiliation, and country locations are believed to be positively associated with research quality. Collaborative work is measured by the number of authors on a publication (*AUTHORS)*. Author affiliation is indicated by four dummy variables, coded as 1 if one of the author affiliations is in academia, industry, government research centers, or hospitals respectively. The count on author affiliations (*AFFILIATIONS*) for an article is included to show if the research involves cross-institution collaboration. Four dummy variables on country location are coded for authors from the US (*US_AUTH*), China (*CHINA_AUTH*), Germany (*GERMANY_AUTH*) and Japan (*JAPAN_AUTH*), the top four countries in nanotechnology publications. The number of author country locations (*AUTH_COUNTRIES*) is counted to indicate whether the article is a product of international collaboration. In addition, research impact measures based on citations are discipline dependent, where certain fields generate more citations than others. A set of 16 field dummy variables is used to control for field disciplinary impacts. When modeling against citations, the effect of the age of an article (*ARTICLE_AGE)* is also controlled since earlier publications have more time to accrue citations than later publications. Our benchmark is December 2010 (the month of data download), and we model the number of months since publication backwards from this time period. A star scientist variable (*STAR_SCIENTIST*) is introduced to control for the influence of the reputation associated with notable scientists. Scientists who are highly cited in the past may tend to receive more citations in the future, in part due to their standing and because their work may be more likely to be accepted into high impact journals, as well as because of the quality of their work. Star scientist is a dummy variable coded as 1 if the article has at least one author with at least 1500 citations (from all WoS papers) in 2000–2007, and 0 if otherwise. Table 3 and Table 4 present the description and summary statistics of all the variables.

**Table 3:** Variable descriptions.

	Variable	Description
Dependent variables	IMPACT_FACTOR	Journal impact factor of the article
	CITATION	Number of citations as of December 2010
Grant sponsorship	FUNDED	1 if article acknowledged funding award support; 0 if not
	FUNDERS	Number of funding sources reported in the article
	FUNDERS2	Squared term of FUNDERS
	FUND_COUNTRIES	Number of funding countries reported in the article
	FUND_COUNTRIES2	Squared term of FUND-COUNTRIES
	EU_FUND	1 if article acknowledged funding support from European Union (EU) programs; 0 if not
	US_FUND	1 if article acknowledged funding support from the US; 0 if not
	CHINA_FUND	1 if article acknowledged funding support from China; 0 if not
	GERMANY_FUND	1 if article acknowledged funding support from Germany; 0 if not
	JAPAN_FUND	1 if article acknowledged funding support from Japan; 0 if not
Articles	ARTICLE_AGE	Age of the article as of December 2010 (in months)
	STAR_SCIENTIST	1 if at least one author is highly cited in 2000–2007; 0 if not
	AUTHORS	Number of authors contributing to the article
	AUTHORS2	Squared term of AUTHORS
Authoraffiliations	ACADEMIC	1 if one author affiliation is university; 0 if not
	CORPORATE	1 if one author affiliation is industry; 0 if not
	GOVLAB	1 if one author affiliation is a government laboratory or public research center; 0 if not
	HOSPITAL	1 if one author affiliation is hospital; 0 if not
	AFFILIATIONS	Number of author affiliations in the article
	AFFILIATIONS2	Squared term of AFFILIATIONS
Author countries	US_AUTH	1 if one author country is the US; 0 if not
	CHINA_AUTH	1 if one author country is China; 0 if not
	GERMANY_AUTH	1 if one author country is Germany; 0 if not
	JAPAN_AUTH	1 if one author country is Japan; 0 if not
	AUTH_COUNTRIES	Number of countries in author affiliations
	AUTH_COUNTRIES2	Squared term of AUTH-COUNTRIES
Field dummies	MULTIMATERIAL	1 if one subject category is multimaterial; 0 if not
	PHYSCHEM	1 if one subject category is physical chemistry; 0 if not
	APPLIEDPHYS	1 if one subject category is applied physics; 0 if not
	MULTICHEM	1 if one subject category is multichemistry; 0 if not
	NANO	1 if one subject category is nanoscience/nanotechnology; 0 if not
	CONDENSPHYS	1 if one subject category is condensed physics; 0 if not
	POLYMER	1 if one subject category is polymer science; 0 if not
	ATOMICPHYS	1 if one subject category is atomic physics; 0 if not
	MULTIPHYSICS	1 if one subject category is multiphysics; 0 if not
	ELECTROCHEM	1 if one subject category is electronic chemistry; 0 if not
	ANALYTICALCHEM	1 if one subject category is analytical chemistry; 0 if not
	BIOCHEMMOLEC	1 if one subject category is biochemical molecule; 0 if not
	INORGANICCHEM	1 if one subject category is inorganic chemistry; 0 if not
	METALLURGY	1 if one subject category is metallurgy; 0 if not
	COATINGS	1 if one subject category is coatings; 0 if not
	OPTICS	1 if one subject category is optics; 0 if not

**Table 4:** Summary statistics of variables.

	Variable	Num of Obs	Mean	Std. Dev.	Min	Max
Dependent variables	IMPACT_FACTOR	89,605	3.242	3.026	0	49.926
	CITATION	89,601	4.674	7.768	0	394
Grant sponsorship	FUNDED	89,605	0.672	0.470	0	1
	FUNDERS	89,605	1.500	1.559	0	34
	FUNDERS2	89,605	4.681	9.904	0	1156
	FUND_COUNTRIES	89,605	0.799	0.679	0	7
	FUND_COUNTRIES2	89,605	1.100	1.677	0	49
	EU_FUND	89,605	0.039	0.194	0	1
	US_FUND	89,605	0.153	0.360	0	1
	CHINA_FUND	89,605	0.160	0.366	0	1
	GERMANY_FUND	89,605	0.043	0.202	0	1
	JAPAN_FUND	89,605	0.042	0.200	0	1
Articles	ARTICLE_AGE	75,002	22.407	3.433	17	28
	STAR_SCIENTIST	89,605	0.017	0.130	0	1
	AUTHORS	89,605	4.745	2.909	1	359
	AUTHORS2	89,605	30.979	454.852	1	128881
AuthorAffiliations	ACADEMIC	89,605	0.868	0.339	0	1
	CORPORATE	89,605	0.078	0.268	0	1
	GOVLAB	89,605	0.368	0.482	0	1
	HOSPITAL	89,605	0.013	0.114	0	1
	AFFILIATIONS	89,605	1.966	1.173	0	54
	AFFILIATIONS2	89,605	5.242	17.203	0	2916
Author Countries	US_AUTH	89,605	0.229	0.420	0	1
	CHINA_AUTH	89,605	0.224	0.417	0	1
	GERMANY_AUTH	89,605	0.086	0.281	0	1
	JAPAN_AUTH	89,605	0.083	0.275	0	1
	AUTH_COUNTRIES	89,605	1.291	0.620	0	20
	AUTH_COUNTRIES2	89,605	2.051	3.678	0	400
Field dummies	MULTIMATERIAL	89,605	0.249	0.432	0	1
	PHYSCHEM	89,605	0.198	0.399	0	1
	APPLIEDPHYS	89,605	0.148	0.356	0	1
	MULTICHEM	89,605	0.143	0.350	0	1
	NANO	89,605	0.115	0.319	0	1
	CONDENSPHYS	89,605	0.102	0.303	0	1
	POLYMER	89,605	0.068	0.251	0	1
	ATOMICPHYS	89,605	0.050	0.217	0	1
	MULTIPHYSICS	89,605	0.036	0.186	0	1
	ELECTROCHEM	89,605	0.037	0.189	0	1
	ANALYTICALCHEM	89,605	0.033	0.180	0	1
	BIOCHEMMOLEC	89,605	0.035	0.183	0	1
	INORGANICCHEMI	89,605	0.033	0.180	0	1
	METALLURGY	89,605	0.033	0.179	0	1
	COATINGS	89,605	0.029	0.169	0	1
	OPTICS	89,605	0.029	0.168	0	1

Source: Analysis of nanotechnology papers, published worldwide August 2008-July 2009, and indexed in the Web of Science (see text for added details). Citations as of December 2010.

To test the hypotheses proposed above, we use two sets of models. In the first set of models, we compare grant sponsored with non-grant sponsored research papers and use a dummy variable (*FUNDED*) showing the status of funding as the key independent variable. In the second set of models, we look at grant sponsored publications only and examine whether the diversity of funding sources has any impact on research quality. To give consideration to the argument that too much collaboration or too many funding organizations might impose added burdens related to administration and communication and thus detract from research quality, we add squared terms to account for quadratic relationships [47]. Count variables for collaboration and funding are tested together with their quadratic counterparts, including the number of authors, the number of author affiliations, the number of author countries, the number of funding sources and the number of funding countries.

In addition, we are interested in exploring whether funding support influences research impact differently for high impact papers or high journal placement papers. A more comprehensive picture of the covariant effects can be obtained by using quantile regression. Quantile regression allows us to see the relationship between independent variables and specific quantiles of the dependent variable [48]. This allows us to examine if funding influence varies among papers in different quantiles of research impact. The four hypotheses are again tested in seven quantiles of journal impact factor and citations. The same set of control variables are used in each of these quantile models.

## Results

In the first set of models, we compare research impact measured by journal impact factor (Model 1) and citation counts (Model 2) with funding status. The dummy variable *FUNDED* is used to indicate whether the paper declares its funding source in the acknowledgment text. The coefficients of this variable are positive and significant in both models. The result shows that publications with funding acknowledgment are more likely to get published in high impact journals and receive more citations thereafter (Table 5). The coefficients in these two models are rather consistent. However, research collaboration exhibits interesting and mixed impacts. While the number of authors contributes to research impact, the number of author affiliations and the number of author countries show U-shaped and inverted U-shaped relationships respectively. According to the coefficients, research impact declines along with the increase of the number of affiliations from 1 to 10, and then increases when the number of affiliations is beyond 10. With the increase of the number of author countries, research impact tends to diminish except in the beginning where authorship from two countries seems to be better than single country authorship. As for other control variables, authorship involving star scientists increases research impact. The type of author affiliation is not a major factor, although papers with corporate authors are less likely to get into high impact journals while those with hospital affiliations are more likely to publish in such journals. It seems that clinical research attracts more citations. Papers published by authors from leading scientific countries garner higher impacts except for authors from China where there are negative coefficients in both models. As expected, the journal impact factor and the age of article both positively influence citations.

**Table 5:** Regression output with funding status as a key independent variable.

	IMPACT_FACTOR	CITATION
Model	(linear regression)	(negative binomial regression)
(1)	(2)
IMPACT_FACTOR		0.192***
		(0.002)
ARTICLE_AGE		0.064***
		(0.001)
FUNDED	0.539***	0.163***
	(0.020)	(0.009)
AUTHORS	0.206***	0.035***
	(0.005)	(0.002)
AUTHORS2	0.000***	0.000***
	(0.000)	(0.000)
STAR_SCIENTIST	1.317***	0.214***
	(0.070)	(0.029)
ACADEMIC	0.025	0.012
	(0.033)	(0.013)
CORPORATE	-0.264***	-0.014
	(0.036)	(0.015)
GOVLAB	0.010	-0.010
	(0.024)	(0.010)
HOSPITAL	0.586***	0.058*
	(0.081)	(0.032)
AFFILIATIONS	-0.061***	-0.030***
	(0.014)	(0.006)
AFFILIATIONS2	0.003***	-0.001*
	(0.001)	(0.000)
US_AUTH	1.233***	0.180***
	(0.023)	(0.010)
CHINA_AUTH	-0.673***	-0.040***
	(0.024)	(0.010)
GERMANY_AUTH	0.714***	0.118***
	(0.034)	(0.014)
JAPAN_AUTH	0.159***	-0.042***
	(0.034)	(0.014)
AUTH_COUNTRIES	0.138***	0.059***
	(0.034)	(0.014)
AUTH_COUNTRIES2	-0.042***	-0.002
	(0.006)	(0.003)
Field dummies	yes	yes
Constant	1.354***	-1.112***
	(0.042)	(0.031)
Num of obs	89,605	75,002
(Adj/Pseudo) R-squared	0.2043	0.072

Source: Analysis of nanotechnology papers, published worldwide August 2008-July 2009, and indexed in the Web of Science (see text for added details). Citations as of December 2010.

Standard errors in parentheses

*** p<0.01,

** p<0.05,

* p<0.1

In the next set of models, we test the relationship between funding diversity and research impact (Table 6). Three types of variables are used to measure funding diversity: the number of funding sources as in *FUNDERS* (Models 3 and 6), the number of funding countries as in *FUND_COUNTRIES* (Models 4 and 7), and funding provided by four leading countries the US, China, Germany and Japan as in *US_FUND*, *CHINA_FUND*, *GERMANY_FUND* and *JAPAN_FUND* (Models 5 and 8). The European Union (EU) as a separate entity also provides various research funding opportunities, including through EU Framework Programmes and the European Network of Excellence. Scientists from European countries are not only eligible for funding support from their own countries, but also from European Union. Therefore we include European Union funding (*EU_FUND)* as a separate funding source to delineate its impact. Funding sources are not mutually exclusive. Overall, the funding diversity variables show a positive impact on research quality with some slight variations. The more funding sources acknowledged in a paper, the more likely the paper will be found to be published in a highly ranked journal. A quadratic relationship is found between the number of funding sources and received citation counts. Citations increase with the number of funding sources, peaks at around 14 sources and then decrease, implying that an optimal level of funding diversity exists for citations. The number of funding countries is found to be positively linked to both impact factor and citations, although nonlinear in terms of the former. The journal impact factor increases at an accelerating pace with the increase of funding countries. Regarding the country origin of funding, the EU, the US, Germany and Japan all appear to have positive impacts on the rank of published journals, but positive effects are retained only for the EU, the US and Germany when it comes to the citation measure. The impact of control variables is found to be consistent with those in Model 1 and Model 2 with the coefficients of control variables very similar to those reported in Table 5.

**Table 6:** Regression output with funding diversity as key independent variables.

Model	IMPACT_FACTOR			CITATION		
		(linear regression)			(negative binomial regression)		
	(3)	(4)	(5)	(6)	(7)	(8)
IMPACT_FACTOR				0.167***	0.168***	0.168***
				(0.002)	(0.002)	(0.002)
ARTICLE_AGE				0.060***	0.060***	0.060***
				(0.001)	(0.001)	(0.001)
FUNDERS	0.259***			0.054***		
	(0.018)			(0.005)		
FUNDERS2	-0.002			-0.002***		
	(0.002)			(0.000)		
FUND_COUNTRIES		0.328***			0.114***	
		(0.102)			(0.036)	
FUND_COUNTRIES2		0.070***			-0.007	
		(0.026)			(0.009)	
EU_FUND			0.700***			0.126***
			(0.052)			(0.019)
US_FUND			0.983***			0.155***
			(0.050)			(0.018)
CHINA_FUND			-0.281***			-0.008
			(0.068)			(0.026)
GERMANY_FUND			0.865***			0.087***
			(0.074)			(0.027)
JAPAN_FUND			0.336***			0.033
			(0.095)			(0.035)
AUTHORS	0.175***	0.189***	0.190***	0.037***	0.039***	0.039***
	(0.006)	(0.006)	(0.006)	(0.002)	(0.002)	(0.002)
AUTHORS2	0.000	0.000	0.000	0.000***	0.000***	0.000***
	(0.000)	(0.000)	(0.000)	(0.000)	(0.000)	(0.000)
STAR_SCIENTIST	1.096***	1.091***	1.053***	0.193***	0.194***	0.188***
	(0.085)	(0.085)	(0.085)	(0.031)	(0.031)	(0.031)
ACADEMIC	-0.023	0.003	-0.009	0.001	0.005	0.004
	(0.044)	(0.044)	(0.044)	(0.016)	(0.016)	(0.016)
CORPORATE	-0.219***	-0.226***	-0.234***	-0.012	-0.011	-0.014
	(0.048)	(0.048)	(0.048)	(0.018)	(0.018)	(0.018)
GOVLAB	-0.031	-0.028	-0.034	-0.010	-0.009	-0.010
	(0.030)	(0.030)	(0.030)	(0.011)	(0.011)	(0.011)
HOSPITAL	0.444***	0.508***	0.542***	0.068*	0.073**	0.080**
	(0.105)	(0.105)	(0.105)	(0.037)	(0.037)	(0.037)
AFFILIATIONS	-0.164***	-0.148***	-0.146***	-0.041***	-0.034***	-0.034***
	(0.022)	(0.022)	(0.022)	(0.008)	(0.008)	(0.008)
AFFILIATIONS2	0.020***	0.021***	0.021***	0.000	0.000	0.000
	(0.002)	(0.002)	(0.002)	(0.001)	(0.001)	(0.001)
US_AUTH	1.227***	1.320***	0.560***	0.175***	0.190***	0.072***
	(0.029)	(0.029)	(0.050)	(0.011)	(0.011)	(0.018)
CHINA_AUTH	-0.708***	-0.575***	-0.254***	-0.033***	-0.009	0.010
	(0.029)	(0.029)	(0.067)	(0.011)	(0.011)	(0.025)
GERMANY_AUTH	0.740***	0.692***	0.098	0.121***	0.114***	0.053**
	(0.044)	(0.044)	(0.067)	(0.016)	(0.016)	(0.025)
JAPAN_AUTH	0.159***	0.154***	-0.063	-0.035**	-0.035**	-0.051
	(0.045)	(0.045)	(0.087)	(0.017)	(0.017)	(0.032)
AUTH_COUNTRIES	0.161***	0.049	0.314***	0.029*	0.006	0.045***
	(0.045)	(0.046)	(0.046)	(0.017)	(0.017)	(0.017)
AUTH_COUNTRIES2	-0.066***	-0.065***	-0.078***	-0.001	0.001	-0.001
	(0.009)	(0.009)	(0.009)	(0.003)	(0.003)	(0.003)
Field dummies	yes	yes	yes	yes	yes	yes
Constant	1.788***	1.811***	1.900***	-0.812***	-0.844***	-0.784***
	(0.059)	(0.093)	(0.056)	(0.037)	(0.045)	(0.037)
Number of obs.	60,202	60,202	60,202	52,407	52,407	52,407
(Adj/Pseudo) R-squared	0.206	0.202	0.205	0.069	0.069	0.069

Source: Analysis of nanotechnology papers, published worldwide August 2008-July 2009, and indexed in the Web of Science (see text for added details). Citations as of December 2010.

Standard errors in parentheses

*** p<0.01,

** p<0.05,

* p<0.1

Using the same set of control variables, the relationship between funding and research impact is tested in quantile regressions. Table 7 and Table 8 report the results for funding status, where the coefficients of control variables are omitted. The impact of funding is much stronger in the upper quantiles of the distribution. For example, the difference in journal impact factor between grant sponsored and non-grant sponsored research publications is 0.24 at the 5^th^ percentile of the conditional distribution and 0.482 at the 75^th^ percentile. Similarly, the disparity between citations of grant sponsored and non-grant sponsored research paper is close to 0 at the 5^th^ quantile and is 0.2 at the 90^th^ quantile. Generally speaking, high impact papers are much more likely to be associated with acknowledged funding compared with low impact papers. However, we also note that the difference is not as big in the very high end of the distribution of journal impact factor (90^th^ and 95^th^ percentiles).

**Table 7:** Quantile regression with funding status on impact factor (N = 89605).

	5th	10th	25th	50th	75th	90th	95th
FUNDED	0.240***	0.326***	0.425***	0.450***	0.482***	0.166***	0.179***
	(0.009)	(0.009)	(0.008)	(0.012)	(0.015)	(0.010)	(0.014)
Pseudo R-squared	0.0822	0.1063	0.1236	0.1726	0.2082	0.2935	0.2831

Source: Analysis of nanotechnology papers, published worldwide August 2008-July 2009, and indexed in the Web of Science (see text for added details). Citations as of December 2010.

Note: Same set of control variables as in Model 1 included in analysis but not reported here.

Standard errors in parentheses

*** p<0.01,

** p<0.05,

* p<0.1

**Table 8:** Quantile regression with funding status on citation (N = 75002).

	5th	10th	25th	50th	75th	90th	95th
FUNDED	0.000*	0.030***	0.111***	0.152***	0.193***	0.200***	0.125
	(0.000)	(0.011)	(0.016)	(0.025)	(0.039)	(0.068)	(0.104)
Pseudo R-squared	0.0000	0.0342	0.1058	0.1752	0.2408	0.3176	0.3716

Source: Analysis of nanotechnology papers, published worldwide August 2008-July 2009, and indexed in the Web of Science (see text for added details). Citations as of December 2010.

Note: Same set of control variables as in Model 2 included in analysis but not reported here.

Standard errors in parentheses

*** p<0.01,

** p<0.05,

* p<0.1

In terms of funding diversity, both the number of funding sources and the number of funding countries show stronger impact in the right tail of the distribution (Tables 9 and 10). The marginal impact of the number of funding organizations is 0.079 at the 5^th^ percentile and 0.198 at the 95^th^ percentile for impact factor, and 0.007 at the 5^th^ percentile and 0.277 at the 95^th^ percentile for citations. Meanwhile, the marginal impact of the number of funding countries increases constantly across percentiles and is tripled at the 95^th^ percentile compared with the 5^th^ percentile for impact factor, and even 30 times at the 95^th^ percentile compared with the 5^th^ percentile for citations. The importance of funding diversity is more evident in high impact articles.

**Table 9:** Quantile regression with funding diversity on impact factor (N = 60202).

	5th	10th	25th	50th	75th	90th	95th
FUNDERS	0.079***	0.081***	0.111***	0.118***	0.122***	0.129***	0.198***
	(0.004)	(0.005)	(0.004)	(0.005)	(0.005)	(0.005)	(0.007)
Pseudo R-squared	0.0812	0.0995	0.1190	0.1724	0.2225	0.3039	0.2631
FUND_COUNTRIES	0.168***	0.201***	0.280***	0.283***	0.253***	0.268***	0.541***
	(0.014)	(0.014)	(0.013)	(0.012)	(0.015)	(0.010)	(0.016)
Pseudo R-squared	0.0780	0.0976	0.1171	0.1707	0.2209	0.3027	0.2616
EU_FUND	0.277***	0.301***	0.400***	0.342***	0.359***	0.209***	0.536***
	(0.028)	(0.025)	(0.021)	(0.025)	(0.028)	(0.027)	(0.026)
US_FUND	0.309***	0.354***	0.488***	0.601***	0.609***	0.460***	1.048***
	(0.028)	(0.024)	(0.021)	(0.024)	(0.026)	(0.026)	(0.023)
CHINA_FUND	-0.053	-0.018	-0.068**	-0.088***	-0.126***	-0.112***	-0.067**
	(0.041)	(0.033)	(0.029)	(0.033)	(0.036)	(0.037)	(0.033)
GERMANY_FUND	0.248***	0.236***	0.308***	0.391***	0.313***	0.313***	0.929***
	(0.041)	(0.035)	(0.031)	(0.036)	(0.039)	(0.039)	(0.037)
JAPAN_FUND	0.190***	0.237***	0.226***	0.217***	0.258***	0.064	0.148***
	(0.055)	(0.047)	(0.039)	(0.046)	(0.049)	(0.049)	(0.046)
Pseudo R-squared	0.0801	0.0995	0.1191	0.1727	0.2229	0.3032	0.2622

Source: Analysis of nanotechnology papers, published worldwide August 2008-July 2009, and indexed in the Web of Science (see text for added details). Citations as of December 2010.

Note: Same set of control variables as in Models 3–5 included but not reported here.

Standard errors in parentheses

*** p<0.01,

** p<0.05,

* p<0.1

**Table 10:** Quantile regression with funding diversity on citation (N = 52407).

	5th	10th	25th	50th	75th	90th	95th
FUNDERS	0.007***	0.026***	0.066***	0.120***	0.176***	0.235***	0.277***
	(0.002)	(0.004)	(0.007)	(0.010)	(0.017)	(0.029)	(0.045)
Pseudo R-squared	0.0044	0.0467	0.1054	0.1696	0.2386	0.3174	0.3709
FUND_COUNTRIES	0.026***	0.084***	0.140***	0.242***	0.336***	0.585***	0.790***
	(0.006)	(0.014)	(0.022)	(0.033)	(0.050)	(0.097)	(0.145)
Pseudo R-squared	0.0044	0.0466	0.1051	0.1692	0.2381	0.3171	0.3707
EU_FUND	0.031***	0.094***	0.156***	0.224***	0.473***	0.402**	0.717***
	(0.012)	(0.027)	(0.044)	(0.059)	(0.098)	(0.183)	(0.274)
US_FUND	0.015	0.083***	0.237***	0.351***	0.581***	0.931***	1.835***
	(0.012)	(0.027)	(0.043)	(0.057)	(0.092)	(0.170)	(0.248)
CHINA_FUND	-0.017	-0.029	0.055	-0.011	0.010	0.232	0.157
	(0.017)	(0.038)	(0.059)	(0.077)	(0.126)	(0.232)	(0.330)
GERMANY_FUND	0.010	0.016	0.140**	0.313***	0.409***	0.437*	0.217
	(0.016)	(0.038)	(0.063)	(0.084)	(0.138)	(0.258)	(0.382)
JAPAN_FUND	-0.026	-0.074	-0.036	-0.062	-0.071	-0.137	0.183
	(0.019)	(0.047)	(0.080)	(0.107)	(0.178)	(0.336)	(0.469)
Pseudo R-squared	0.0044	0.0466	0.1051	0.1692	0.2382	0.3171	0.3712

Source: Analysis of nanotechnology papers, published worldwide August 2008-July 2009, and indexed in the Web of Science (see text for added details). Citations as of December 2010.

Note: Same set of control variables as in Models 6–8 included but not reported here.

Standard errors in parentheses

*** p<0.01,

** p<0.05,

* p<0.1

Funding from selected leading countries shows its role in improving research impact, which also increases across quantiles. The impact of funding from the EU, the US, Germany and Japan is higher on the upper quantiles of the distribution of impact factor. For citation counts, only EU funding and US funding are mostly significant in the whole range of the distribution. Similarly, the disparity between publications received funding from these two sources and those without is much higher in the right tail of the distribution.

## Conclusion and Discussion

The study explored how funding affects research impacts. It is among the first set of studies that text mine funding acknowledgment in WoS records to systematically examine the relationship between funding and impact at a large scale. Our analysis of the funding acknowledgment section of nanotechnology publications in 2008–2009 finds that outputs from grant sponsored research exhibit higher impacts than outputs from non-grant sponsored research. Grant sponsored articles are not only more likely to get published in highly ranked journals, but also to generate more research interest in the field as measured by forward citations. The diversity of funding sources has a more variable influence on research impact. The number of funding sources acknowledged in publications is positive on placement in high quality journals, but tends to be concave on received citations, increasing before reaching the optimal number of funding sources, and then decreasing. This suggests that the quality assurance of multiple review processes is effective to some extent but also that too many funding entities may increase transactional burdens and distract from the research itself. The number of funding countries contributes to both journal placement and citation counts. Research supported by funding from multiple countries does imply international value and potential, which may make it more acceptable to peer reviewers and the research community. Research grants provided by selected leading countries/blocs such as the EU, the US and Germany are even more influential. Publications indicating financial support from these countries/blocs appear more often in good journals and receive more attention through citations. It is plausible that research funded by these countries is of higher quality, although other factors may also be at work. For example, it is possible that research priorities funded by leading countries shapes research directions in other countries and thus generates recognition and follower citations.

In addition, according to the quantile regression results, grant funding appears to have stronger influence in the production of high impact research publications. This suggests that the grant review process does select more promising projects, since it is at the higher ends of impact where the disparity between grant sponsored and non-grant sponsored research is most substantial. To some extent, this finding validates the use of grant funding mechanisms to allocate R&D funds, although we emphasize that this result should be interpreted cautiously. There is some circularity in the process: more motivated and innovative researchers are more likely to get grants and in turn produce research that achieves higher impact. This is a positive cyclic process. Those who are not able to break into this cycle may face difficulties in an increasingly competitive research world.

There are caveats that are relevant to this study. On the one hand, not all grant funding is acknowledged. Some might be undisclosed intentionally or simply neglected. On the other hand, not all acknowledged funding is acquired through competitive peer-reviewed process. Some may be earmarked or supported by institutional funds. Therefore, acknowledged funding cannot be equated with competitive grant funding in every case. Nevertheless, given the high correlation between grant funding and publication acknowledgment [26] [45], we judge that this limitation does not overly bias the results. As discussed in the paper, we acknowledge that there are limitations to the use of journal impact factors and citation counts to measure research quality.

This study seeks to untangle the relationships between grant funding and research impact. It provides empirical evidence of the effectiveness of grant funding schemes and the scientific publication impacts of different combinations of research investments. The study suggests that research collaboration is beneficial not only by bringing together different skills and mindsets but also by coupling financial resources to produce research outputs that generate higher publication impacts. As for policy implications, the study suggests that joint solicitation for research proposals by different funding agencies is a productive way to promote effective research partnerships. Research funding agencies may also find it productive to encourage more international collaborative research activities by their home scientists and to actively collaborate with counterparts in other countries.
